# Supplementary material for: Deletion of wfs1 Impairs Oligodendrocyte Precursor Cells Dorsal Distribution and Myelination Through the wfs1-hmgcs1 Axis in Zebrafish
Source: Biology (Basel). 2026 Aug 16;15(16):1402. doi: 10.3390/biology15161402 (PMC13510151; doi:10.3390/biology15161402)
Supplement: Supplementary file 1 [file biology-15-01402-s001.zip › File S1-Transcriptome sequencing data and differentially expressed genes (DEGs) in wfs1-- zebrafish at 5 dpf/2.KEGG/wfs1KOvsWT/DOWN/src/dre00562.html]

dre00562


- dre:337236

- Down regulated genes

337236\_1(-2.86219060000093)

- dre:368717

- Down regulated genes

368717\_1(-1.74128930765284)

- dre:559198

- Down regulated genes

559198\_1(-1.12263541156314)

- dre:566479

- Down regulated genes

566479\_1(-1.58815130182225)

- dre:560753

- Down regulated genes

560753(-1.42485234049394)

- dre:571356

- Down regulated genes

571356\_1(-1.36763102903653)

- dre:559198

- Down regulated genes

559198\_1(-1.12263541156314)

Close
